# The graft-versus-leukemia effect of prophylactic donor lymphocyte infusions after allogeneic stem cell transplantation is equally effective in relapse prevention but safer compared to spontaneous graft-versus-host disease

**DOI:** 10.1007/s00277-023-05276-5

**Published:** 2023-07-25

**Authors:** Michael Stadler, Lothar Hambach, Elke Dammann, Helmut Diedrich, Haytham Kamal, Iyas Hamwi, Christian Schultze-Florey, Michael Varvenne, Steve Ehrlich, Stefanie Buchholz, Christian Koenecke, Gernot Beutel, Eva M. Weissinger, Jürgen Krauter, Matthias Eder, Bernd Hertenstein, Arnold Ganser

**Affiliations:** Department of Hematology, Hemostasis, Oncology, and Stem Cell Transplantation, Hannover Medical School, Carl-Neuberg-Str. 1 , D – 30625 Hannover, Germany

**Keywords:** Prophylactic donor lymphocyte infusion, Allogeneic stem cell transplantation, Acute leukemia, Myelodysplastic syndrome, Graft-versus-leukemia, Graft-versus-host

## Abstract

Therapeutic donor lymphocyte infusions (tDLI) are used to reinforce the graft-versus-leukemia (GvL) effect in relapse after allogeneic stem cell transplantation (alloSCT). In contrast, the role of prophylactic DLI (proDLI) in preventing leukemia relapse has been less clearly established, although supported by retrospective, case-control, and registry analyses. We report a prospective, monocentric, ten year cohort of patients with high risk acute leukemias (AL) or myelodysplasia (MDS) in whom proDLI were applied beyond day +120 post alloSCT to compensate for lack of GvL.

272 consecutive allotransplanted AL or MDS patients in complete remission and off immunosuppression at day +120 were stratified according to the prior appearance of relevant GvHD (acute GvHD °II-IV or extensive chronic GvHD) as a clinical indicator for GvL. Escalating doses of unmodified proDLI were applied to 72/272 patients without prior relevant GvHD. Conversely, 157/272 patients with prior spontaneous GvHD did not receive proDLI, nor did 43/272 patients with contraindications (uncontrolled infections, patient refusal, DLI unavailability).

By day 160-landmark analysis (median day of first DLI application), proDLI recipients had significantly higher five-year overall (OS) and disease free survival (DFS) (77% and 67%) than patients with spontaneous GvHD (54% and 53%) or with contraindications (46% and 45%) (p=0.003). Relapse incidence for patients with proDLI (30%) or spontaneous GvHD (29%) was significantly lower than in patients with contraindications (39%; p=0.021). With similar GvHD incidence beyond day +160, non-relapse mortality (NRM) was less with proDLI (5%) than without proDLI (18%; p=0.036).

In conclusion, proDLI may be able to compensate for lack of GvL in alloSCT recipients with high risk AL or MDS.

**Supplementary Information:**

The online version contains supplementary material available at 10.1007/s00277-023-05276-5.

## Introduction

Allogeneic stem cell transplantation (alloSCT) can cure acute leukemias (AL), myelodysplastic syndromes (MDS), and other hematologic malignancies, mainly through donor-versus-recipient alloreactivity. This graft-versus-leukemia (GvL) or graft-versus-malignancy (GvM) effect, however, cannot yet be predicted or even reliably quantified, leaving the about 50% of allotransplanted patients without clinically evident graft-versus-host disease (GvHD) in the uncertainty of a potentially increased relapse risk (apart from other known factors of relapse risk such as genetic profile of the disease, remission status at transplantation, donor-recipient match, conditioning regimen, and immunosuppression). Indeed, relapse is the leading cause for failure of alloSCT in hematologic malignancies, and, once established, has a severely impaired outcome [1], even with post-transplant immune interventions [2]. Since mild GvHD is associated with improved protection against relapse [3], we reasoned that high risk leukemia patients without GvHD after withdrawal of immunosuppression should be given prophylactic donor lymphocyte infusions (proDLI) in order to compensate for lack of a perceptible GvL effect. These proDLI should be performed in escalating doses to lower the risk of DLI-induced GvHD [4].

Donor lymphocyte infusions (DLI) have been widely employed as a method to reduce relapse probability [5]. Whereas *therapeutic* DLI (tDLI) are used in overt relapse and *preemptive* DLI (preDLI) in response to declining donor chimerism or emerging residual disease, the application of truly *prophylactic* DLI (proDLI) is solely based on the presence of established risk factors for malignant relapse. PreDLI administered before day +100 after alloSCT, especially with *in-vitro* T-cell-depleted transplants, have been shown to convert mixed to full donor chimerism, however, often at the expense of excessive GvHD [6, 10], unless CD8-depleted [11]. G-CSF stimulated (“modified”) proDLI, concurrently with short-term immunosuppression, have been used very early after matched or mismatched/haploidentical transplantation [12].

The use of unmodified, unmanipulated proDLI after alloSCT without *in-vitro* T-cell-depletion, in order to induce GvL in patients lacking spontaneous GvHD, was pioneered by the Munich group in FLAMSA-based sequential conditioning protocols for AML [13], but also recommended for ALL [14]. Surprisingly, there have been only few published data to support this important approach: in prospective analyses, Schmid et al. [13] described 17 AML patients receiving proDLI with a 2-year overall survival (OS) of 87%; Legrand et al. [15] reported 8 proDLI patients with 76% OS at 2 years. Two retrospective case-control studies opposing 41 patents with proDLI versus 35 matched controls [16] and 27 patients with proDLI versus 23 controls [17], respectively, demonstrated improved OS in high risk AML patients. For the use of proDLI in ALL, proof of principle has been provided by a cohort of 15 patents [18]. Finally, the EBMT registry collected 96 AML and 30 ALL patients having received proDLI [19].

To this evidence, we want to contribute long-term results of 72 recipients of proDLI within our prospective cohort of 272 consecutive high risk AL or MDS patients allotransplanted without *in-vitro* T-cell-depletion between July 2004 and March 2013, to whom we systematically offered proDLI after day +120 post alloSCT, if they had not experienced clinically relevant spontaneous GvHD before.

## Patients and Procedures

### Design and cohort definition

This is the post-hoc analysis of a prospective cohort. Starting from July 2004, we adopted as our institutional policy to systematically offer proDLI to all consecutive eligible patients with AL or MDS at high risk of relapse after a first alloSCT without *in-vitro* T-cell-depletion, and lacking clinically relevant spontaneous GvHD until day +120, as recommended by FLAMSA based protocols for *de novo* or secondary AML and MDS [13, 20], and the German Multicenter ALL (GMALL) protocol for ALL [14]. In these protocols, immunosuppression is tapered until day +90 after alloSCT in order to allow for the development of a GvL effect; proDLI are recommended for patients without GvHD until day +120. High risk criteria (evaluated at the time of alloSCT) included adverse cytogenetics or molecular genetics (reflecting European LeukemiaNet criteria [21]), relapsed or refractory disease, extramedullary or therapy-related AL, ALL with measurable residual disease after consolidation chemotherapy, pre-B-ALL, mature T-ALL, or MDS with excess of blasts.

Excluded were patients with *in-vitro* T-cell-depleted grafts, haploidentical, cord blood, syngeneic or second allogeneic transplantation, participants in other transplant studies with OS or DFS as endpoints [22, 23], as well as patients with mixed chimerism or molecular or clinical relapse until day +120 after alloSCT.

Patients with full donor chimerism and WHO performance score 0 or 1 at day +120 were stratified according to the absence or presence of prior clinically relevant GvHD (i.e., acute GvHD grade II to IV [24], or extensive chronic GvHD [25]), to receive or not to receive proDLI, respectively, unless contraindicated. Contraindications against proDLI included: uncontrolled infectious complications, patient’s refusal or DLI unavailability.

Data evaluation was in accordance with the declaration of Helsinki. All patients had given written informed consent to treatment, data analysis and publication prior to alloSCT. Additionally, every eligible patient was asked independently for informed consent prior to proDLI. Evaluation was approved by the institutional ethics committee (10263_BO_K_2022).

### Prophylactic Donor Lymphocyte Infusions (proDLI)

proDLI procedures have been described in the FLAMSA-based protocols [13, 20]. Briefly, proDLI were performed after immunosuppression had been withdrawn for at least 4 weeks, only in eligible patients without prior clinically relevant GvHD and without uncontrolled infectious complications. We started proDLI as soon as possible after day +120 and no later than one year post alloSCT. Unstimulated donor peripheral blood leukaphereses were performed without *in vitro* manipulation. proDLI were started at a dose of 5 x 10E5 (for matched unrelated or mismatched donors) or 1 x 10E6 (in case of matched related donors) CD3 positive donor cells per kg of recipient’s weight, and were escalated in two steps with at least monthly intervals up to 5 x 10E6 and 1 x 10E7 CD3 positive cells per kg, unless symptoms or signs of GvHD occurred. First proDLI doses were usually administered freshly after leukapheresis. Aliquots of donor lymphocytes were cryopreserved and thawed immediately prior to subsequent proDLI. Premedication consisted of antihistamines, but no steroids. After proDLI administration, patients were monitored by outpatient clinical visits every two weeks.

### Statistical analysis

Survival curves were calculated according to the method of KAPLAN & MEIER [26]. Primary (overall survival) and secondary endpoints (disease-free survival, relapse incidence, non relapse mortality, and GvHD incidence after proDLI) were evaluated in landmark analyses from day +160 after alloSCT (the median day of first proDLI application), to reduce lead time bias of day 120-landmark analyses. However, outcome differences between these landmark points were small (Suppl. Table 2). Prognostic factors for OS and DFS were examined by univariate analysis employing Cox regression for continuous covariates and Logrank test for categorical covariates. Multivariate analysis of covariates was performed by forward stepwise Cox regression using current SPSS software.

## Results

### Patients’ cohort

**Fig. 1:**
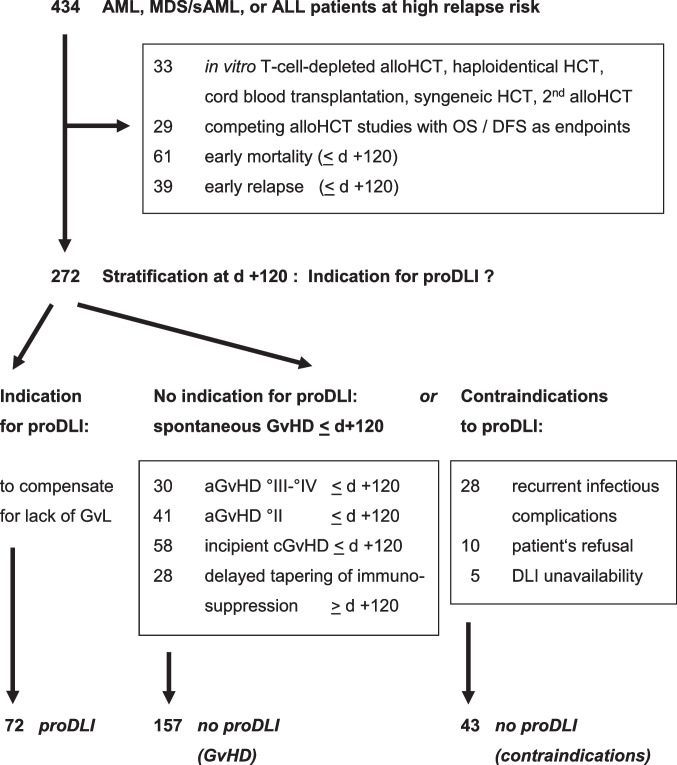
Flow chart

Out of 434 patients with high risk AL or MDS receiving an alloSCT without *in-vitro* T-cell-depletion from July 2004 to March 2013 at our institution, 272 matched the inclusion criteria (Fig. 1). All eligible patients with ALL (n=35) had been transplanted according to the current GMALL protocol [14], most patients with *de novo* AML (n=132) or MDS / secondary AML (n=105) according to the FLAMSA [13] or FLAMSA-based ClAraC [20] protocols; some elderly patients had received conditioning with BCNU/Fludarabin/Melphalan (BFM) [27].

Patients’ characteristics are listed in Table 1.

**Table 1:** Patients’ characteristics

Characteristic	Total Cohort	proDLI	no proDLI for spont. GvHD	no proDLI for contraindications	*p*
Number	272	72	157	43	
Gender:					*0.212*
Female	124 *(46%)*	39 *(54%)*	68 *(43%)*	17 *(40%)*	
Male	148 *(54%)*	33 *(46%)*	89 *(57%)*	26 *(60%)*	
Age:					*0.546*
< 55 years	161 *(59%)*	39 *(54%)*	97 *(62%)*	25 *(58%)*	
> 55 years	111 *(41%)*	33 *(46%)*	60 *(38%)*	18 *(42%)*	
Diagnoses:					*0.410*
*de novo* AML	132 *(48%)*	31 *(43%)*	83 *(53%)*	18 *(42%)*	
MDS/sAML	105 *(39%)*	33 *(46%)*	53 *(34%)*	19 *(44%)*	
ALL	35 *(13%)*	8 *(11%)*	21 *(13%)*	6 *(14%)*	
Karyotype:					*0.510*
Complex	64 *(24%)*	17 *(24%)*	40 *(26%)*	7 *(16%)*	
Other abnormal	102 *(37%)*	24 *(33%)*	62 *(39%)*	16 *(37%)*	
Normal	98 *(36%)*	28 *(39%)*	52 *(33%)*	18 *(42%)*	
Unknown	8 *(3%)*	3 *(4%)*	3 *(2%)*	2 *(5%)*	
Remission at alloHCT:					*0.345*
CR1	93 *(34%)*	28 *(39%)*	54 *(34%)*	11 *(26%)*	
Other	179 *(66%)*	44 *(61%)*	103 *(66%)*	32 *(74%)*	
Conditioning:					*0.092*
Myeloablative	78 *(29%)*	15 *(21%)*	53 *(34%)*	10 *(23%)*	
Reduced Intensity	194 *(71%)*	57 *(79%)*	104 *(66%)*	33 *(77%)*	
Donor match:					*0.344*
Matched related	70 *(26%)*	22 *(31%)*	40 *(26%)*	8 *(19%)*	
Matched unrelated	147 *(54%)*	34 *(47%)*	88 *(56%)*	22 *(51%)*	
Mismatched	55 *(20%)*	16 *(22%)*	29 *(18%)*	13 *(30%)*	

Abbreviations: *ALL *Acute Lymphoblastic Leukemia, *AML *Acute Myeloid Leukemia, *CR1 *first complete remission, *GvHD *Graft-versus-Host Disease, *MDS/sAML *Myelodysplastic Syndrome / secondary AML, *proDLI *prophylactic donor lymphocyte infusions

### ProDLI administration

72 patients (31 AML, 33 MDS/sAML, 8 ALL) who had not experienced clinically relevant GvHD after withdrawal of immunosuppression, were treated with proDLI (median start day +160; range: days +121 to +359). proDLI were not given to the other 200 eligible high risk AL patients: 157 did not receive proDLI for GvHD-associated reasons: 71 acute GvHD grade II to IV until day +120, 58 extensive chronic GvHD until day +120, 28 inability to taper immunosuppression until day +120 (suggesting an ongoing subclinical graft-versus-host reaction). 43 did not receive proDLI for contraindications (28 recurrent infectious complications, 10 patient refusal, 5 DLI unavailability) (Fig. 1). The three groups were well balanced regarding gender, age, diagnosis, karyotype, remission status at alloSCT, conditioning intensity, and donor match (Table 1).

In total, 168 proDLIs were administered to 72 patients with a median of 3 proDLI doses per patient (range: 1-3). 37 patients received all three scheduled proDLI doses. 22 patients received only two proDLI and 13 patients only one, due to ensuing symptoms or signs of incipient GvHD. No relevant acute toxicity of the proDLI administration itself was noted.

### Graft-versus-host disease and non relapse mortality

After proDLI, no acute GvHD grade IV occurred in our cohort. After the first proDLI, there was no acute GvHD beyond grade I. With a median of four weeks after the second proDLI, acute GvHD grade II to III occurred in 4/72 patients (6%) who all responded to standard immunosuppressive treatment, but of whom one patient later progressed to extensive chronic GvHD. Overall, extensive chronic GvHD developed in 11/72 patients (15%), 2 to 23 (median: 3) months after the second or third proDLI. Two of these patients died without evidence of malignant relapse (one from progressive pulmonary GvHD, one from pneumonia). The resulting GvHD-related proDLI mortality was below 3% in our cohort. Three other patients died without evidence of malignant relapse, from complications not attributable to GvHD (one from a cranial trauma, 3 months after proDLI; the other two from second cancers, both more than 5 years after proDLI).

**Fig. 2:**
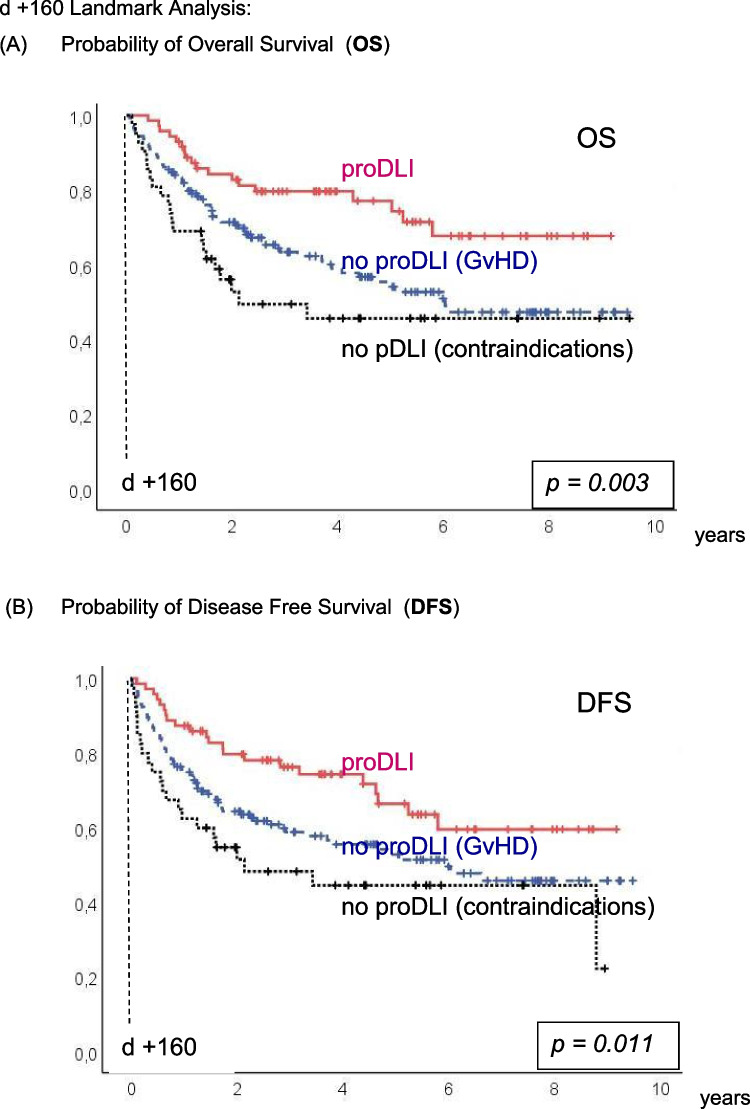

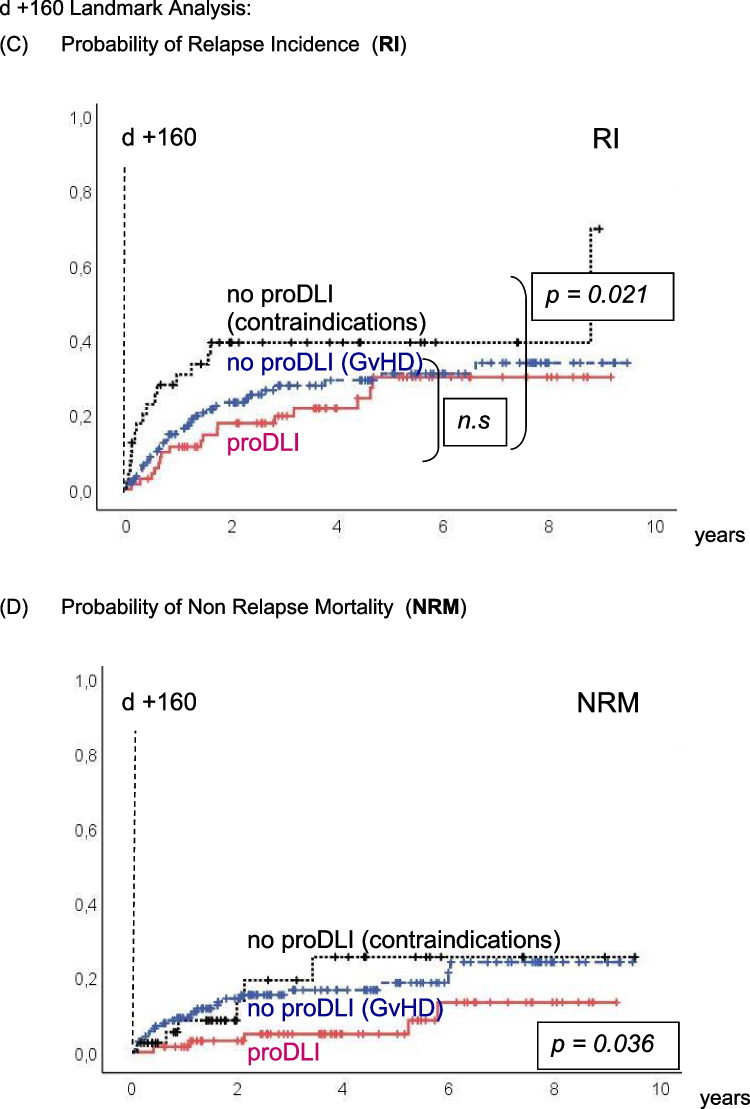

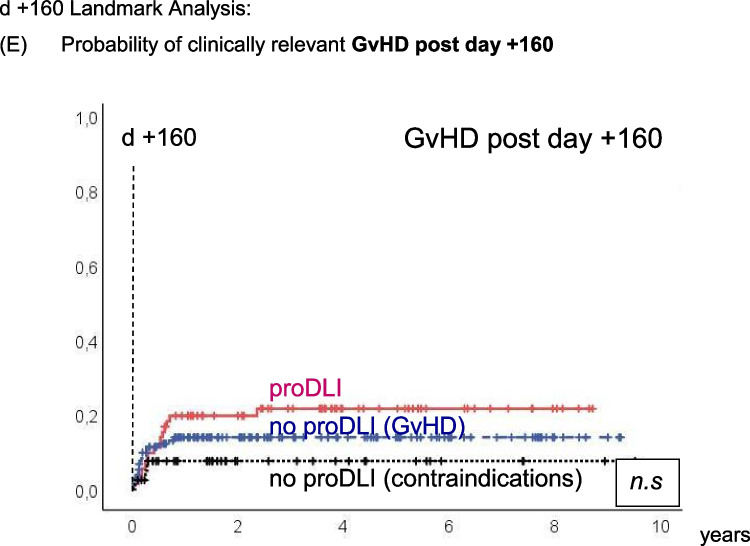
d +160 Landmark Analysis: Outcome of proDLI *versus* no proDLI (GvHD) *versus* no proDLI (contraindications): **A** Probability of Overall Survival (OS). **B** Probability of Disease Free Survival (DFS). **C** Probability of Relapse Incidence (RI). **D** Probability of Non-Relapse Mortality (NRM). **E** Probability of clinically relevant GvHD post day +160

The incidence of clinically relevant subsequent GvHD after day +160 (median day of first proDLI) was not significantly different in proDLI patients (22%) compared to patients not having received any proDLI (12%) (Fig. 2E), even when compared only to the subgroup of patients not having received any proDLI for contraindications (7%, p=0.108). However, non relapse mortality (NRM) after day +160 was significantly lower in proDLI patients (5%) than in patients not having received any proDLI (18%): p=0.036 (Fig. 2D).

### Disease relapse

In 18/72 patients, disease relapse occurred despite proDLI, with a median interval of 15 (range: 1 to 54) months after the last proDLI administration. The resulting relapse incidence (RI) of 30% was similar to patients not given proDLI for prior spontaneous GvHD (29%), but significantly reduced compared to the patients not having received proDLI for contraindications (39%; p=0.021) (Fig. 2C). Despite subsequent chemotherapy, therapeutic DLI, and/or second transplantation), 13/18 (72%) relapsed patients died. Still, durable remissions were again achieved in 5/18 (28%) relapsed patients.

### Survival

Figure 2A and B show the KAPLAN-MEIER curves for OS and DFS, respectively.

By day-160-landmark analysis, five-year OS of proDLI patients was 77%, compared to 54% in patients without proDLI due to prior spontaneous GvHD, and 46% in patients without proDLI for other reasons (p=0.003). Respective five-year DFS of proDLI patients was 67%, compared to 53% in patients without proDLI due to prior spontaneous GvHD, and 45% in patients without proDLI for contraindications (p =0.011).

### Subgroup analyses

#### Subgroups according to diagnoses de novo AML, MDS/sAML, or ALL

**Fig. 3:**
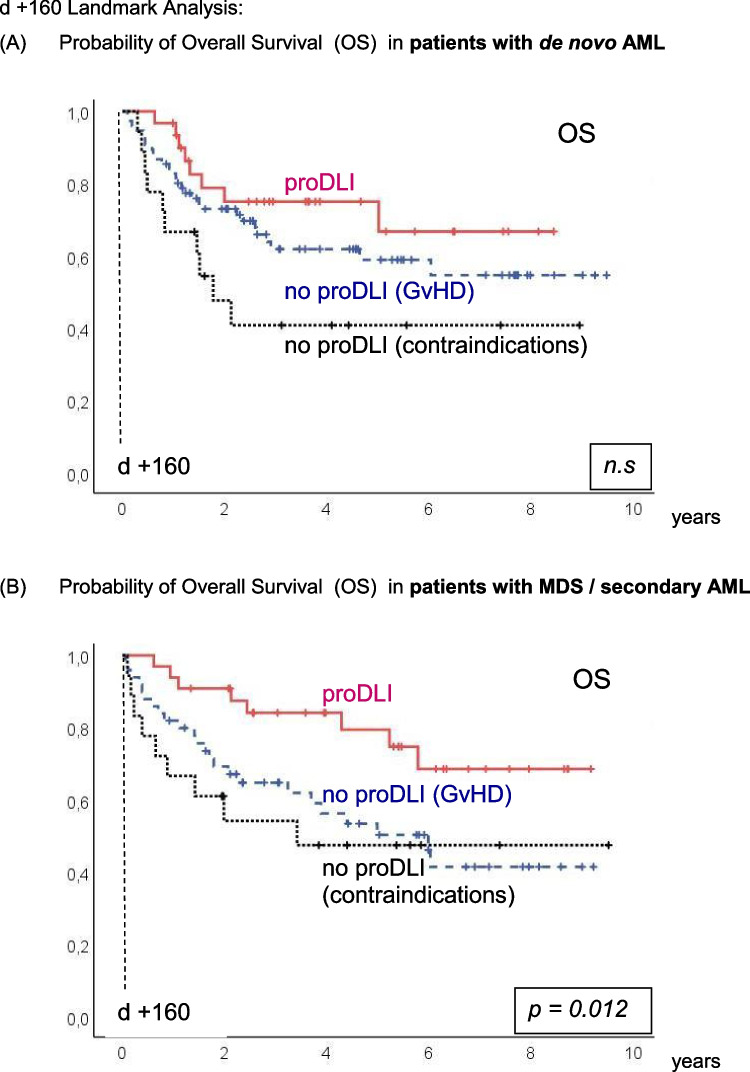

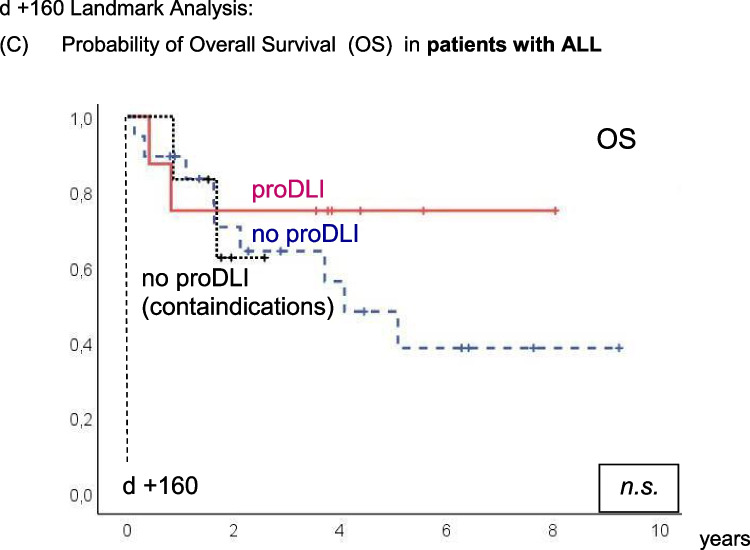
d +160 Landmark Analysis: Probability of Overall Survival (OS) in patients with. **A** *de novo* AML. **B** MDS / secondary AML. **C** ALL

The advantage with regard to five-year OS of proDLI versus no proDLI was as follows for the diagnosis subgroups: in *de novo* AML, 75% versus 56%; in MDS/sAML, 80% versus 50%; and in ALL, 75% versus 44% (Fig. 3A-C). However, these differences reached statistical significance only in MDS/sAML (p=0.012) and a trend in *de novo* AML (p=0.134), but not in ALL patients, likely due to limited patient numbers (8 with pDLI versus 25 without pDLI). Alternatively, the rather slow GvL-effect may conceivably be more effective in diseases with low growth kinetics like MDS/sAML.

#### Number of proDLI doses

**Fig. 4:**
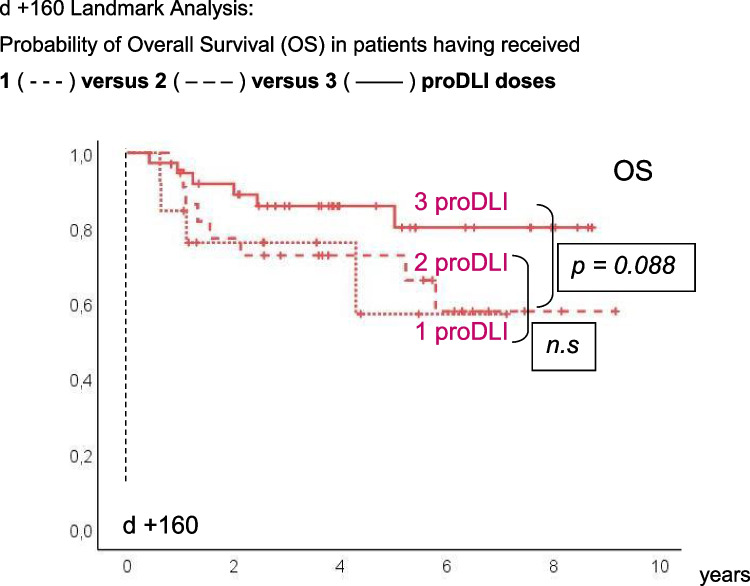
d +160 Landmark Analysis: Probability of Overall Survival (OS) in patients having received. 1 ( - - - ) versus 2 ( – – – ) versus 3 ( –––– ) proDLI doses

Five-year OS, DFS, RI, and NRM showed no significant differences between patients having received 1 versus 2 versus 3 proDLI doses, possibly due to limited patient numbers. However, there was a trend towards superior five-year OS in proDLI patients having received the final dose of 1 x 10E7 CD3 positive donor cells per kg of recipient’s weight (86%), when compared to the combined groups of patients with only 1 or 2 smaller proDLI doses (68%; p=0.088) (Fig. 4).

#### Patients without proDLI for non-severe GvHD

To exclude a bias against patients with possibly inferior outcome due to severe GvHD before d +120, we evaluated whether the advantage of proDLI patients held also true versus patients without proDLI for prior non-severe GvHD only (acute GvHD grade II or limited chronic GvHD). Even compared to this group which should benefit from improved protection against relapse without the disadvantages of severe GvHD [3], patients with proDLI showed significantly better OS (p=0.005), DFS (p=0.016), and RI (p=0.033) (Suppl. Figure 5).

#### Patients without subsequent clinically relevant GvHD

We wondered whether the manifestation of significant GvHD was required for patients after proDLI to maintain the outcome advantage compared to patients without proDLI. When considering only patients without subsequent clinically relevant GvHD (i.e., acute GvHD grade II to IV, or more than limited chronic GvHD) after day +160, OS (p=0.011), DFS (p=0.031), RI (p=0.040), and NRM (p=0.012) were still superior in the proDLI group, suggesting that clinically relevant GvHD after proDLI is not necessary to ascertain “enough” GvL protection. (Suppl. Figure 6).

### Analysis of predictive factors for OS, DFS, RI, NRM, and GvHD in proDLI recipients

To determine factors predictive for the success of proDLI administration, we evaluated in univariate analysis subgroups of patients with complex karyotype, chromosome 7 abnormalities outside the context of a complex karyotype, normal karyotype, internal tandem duplication of the fms-like tyrosine kinase 3 (FLT3-ITD), mutation of the tyrosine kinase domain of the fms-like tyrosine kinase 3 (FLT3-TKD), rearrangement of the mixed lineage leukemia (MLL) gene, or extramedullary disease. Compared to patients without proDLI, recipients of proDLI showed significant five-year OS, DFS, and RI advantages by Cox regression in the subgroups with normal karyotype: OS 100%, p<0.001; DFS 82%, p=0.011; RI 18%, p=0.031; (NRM 0%, not significant); (Suppl. Figure 7) and in FLT3-ITD positive patients: OS 100%, p = 0.001; DFS 100%, p = 0.002; RI 0%, p = 0.014; (NRM 0%, not significant); (Suppl. Figure 8).

To investigate factors predictive for acute or chronic GvHD post proDLI, we analyzed donor type (related versus unrelated; 10/10 versus 9/10 match) and donor gender (female donor for male recipient versus all other combinations) in proDLI recipients. Only the presence of a mismatched donor was significantly predictive for the development of acute GvHD post proDLI (p=0.032); however, the lack of any other statistical association may also be due to limited patient numbers.

### Multivariate analysis of prognostic factors

To analyze the prognostic impact of established patient and transplant risk factors in comparison to proDLI application, we performed Cox regression on the whole cohort of 272 patients, entering gender, age, diagnosis (AML, MDS/sAML, or ALL), karyotype (complex, other abnormal, or normal), presence or absence of complete remission at alloSCT, myeloablative or reduced intensity conditioning, donor match (matched related, matched unrelated, or mismatched), and proDLI application into the model in a forward stepwise manner. For OS, the only significant factors in the model were: *proDLI application* (Hazard ratio, HR: 0.351; 95% Confidence Interval, 95%CI: 0.206-0.598; p<0.001), *age* (HR: 1.024; 95%CI: 1.009-1.040; p=0.002) and *complex karyotype* (HR: 1.711; 95%CI: 1.144-2.561; p=0.009). For DFS, the only significant factors in the model were: *proDLI application* (HR: 0.435; 95%CI: 0.271-0.699; p=0.001), *complex karyotype* (HR: 1.733; 95%CI: 1.183-2.537; p=0.005) and *age* (HR: 1.017; 95%CI: 1.003-1.032; p=0.017). Thus, application of proDLI was the strongest prognostic factor for both OS and DFS.

## Discussion

The graft-versus-leukemia (GvL) effect [28], established through animal studies in the 1950s to 1970s, is held responsible for the observed lower relapse rates in allogeneic versus syngeneic transplantation [29], alloSCT with GvHD versus without GvHD [30], and T-cell-replete versus T-cell-depleted alloSCT [31]. Control or eradication of the malignancy through GvL is believed to represent the mechanism of cure in alloSCT. However, as there are no technical possibilities yet to predict, quantify or “dose” GvL in humans, it remains a matter of chance (and perhaps clinical skill), how much of the potential donor-versus-recipient alloreactivity will become effective in a given patient, and whether its benefits can be balanced against its downside, i.e. severe GvHD.

We reasoned that patients with high risk AL or MDS lacking clinically relevant GvHD after alloSCT (as a raw measure for donor-versus-recipient alloreactivity undergone) be considered candidates for careful post-transplant immune interventions such as early tapering of immunosuppression and escalating proDLI, which we therefore systematically offered to them as our institutional policy since July 2004. Since for ethical and practical reasons, randomized studies of proDLI are unlikely to be feasible, we instead used a “biological” stratification at day 120 according to the presence or absence of clinically relevant previous alloimmune reactions to guide the necessity of additional immune interventions. We made every effort to exclude selection bias by prospective screening of all our consecutive high risk AL or MDS patients between July 2004 and March 2013, resulting in well balanced comparator groups. Setting out to evaluate proDLI as a means for compensation of missing alloreactivity, we would have expected, if anything, an excess of proDLI-induced GvHD. Instead, proDLI proved to elicit only limited subsequent GvHD complications (no grade IV acute GvHD, GvHD-related mortality below 3%) resulting in a significant reduction of NRM. By landmark analyses at day 160 (median day of first proDLI) we found a relapse-protective, induced GvL effect of proDLI comparable to spontaneous GvHD, but with less NRM through subsequent GvHD, leading to superior OS and DFS in proDLI recipients. The observed lower NRM could not be attributed to performance status, since WHO scores 0 or 1 were required for all eligible patients. Nor was it due to a bias against patients with prior severe GvHD in the comparator group, because even in patients with prior *non-severe* GvHD only, OS, DFS, and NRM remained favorable in proDLI recipients. The observed GvL effect was also operative in proDLI patients without subsequent clinically relevant GvHD. There was a trend towards superior five-year OS in proDLI patients having received the final dose of 1 x 10E7 CD3 positive donor cells per kg of recipient’s weight (which also represents the recommended “starting dose” of *therapeutic* DLI), as compared to the smaller doses only – possibly indicating a dose relationship for improved GvL. Especially patients with a normal karyotype or with an FLT3-ITD [32] seemed to benefit from relapse prevention through proDLI. In multivariate analysis, application of proDLI was the most important prognostic factor for OS and DFS.

In our interpretation, this structured approach with slow dose increases and frequent outpatient monitoring allowed for a more carefully “dosed” alloreactivity compared to the imponderables of spontaneous GvHD. It is easily applicable in a clinical outpatient setting, requiring no specialized laboratory or molecular testing procedures. However, it might also be improved by steering tools such as highly sensitive monitoring of MRD [33] or recipient chimerism [34, 35], as well as methods for early detection of proDLI-induced GvHD, e.g. by analysis of GvHD specific proteomic patterns [36, 37], biomarkers [38], or a wide array of recently developed techniques to separate GvHD from GvL [39]. Furthermore, the anti-leukemic efficacy of proDLI might be enhanced by concomitant application of minor histocompatibility antigen based vaccines [40] or by depletion of regulatory T cells [41]. Conversely, depletion of CD45RA positive naïve T cells has been shown to limit GvHD after proDLI [42]. Finally, safety of proDLI might be improved by transduction of suicide genes [43, 44].

However, this approach provides no solution for very early AL relapses occurring in the first five months post alloSCT, respectively before a proDLI-induced GvL effect could technically become effective. Also, very high risk patients (e.g. with complex karyotype, MLL rearrangement, or extramedullary disease) did not seem to benefit from proDLI in our exploratory analysis of predictive factors. Future studies might address the possibility of even earlier tapering of immunosuppression and administration of proDLI in these subgroups of patients.

Although proDLI seemed to provide long lasting protection in the majority of our cohort, our knowledge about the durability of such induced GvL effects is limited, Relapses occurring despite proDLI in up to one fourth of patients, in single cases even beyond 4 years after proDLI, raise the question whether late tolerance might eventually abrogate the initial GvL activity, and whether proDLI, perhaps without dose increase, could be repeated in regular intervals [45].

To conclude, in high risk acute leukemia or myelodysplasia patients lacking clinically relevant GvHD after alloSCT without *in-vitro* T-cell-depletion, unmodified proDLI after day +120 are feasible, safe and effective, reduce relapse incidence comparably to spontaneous GvHD, without increasing non-relapse mortality, and seem to induce a more carefully “dosed“ GvL effect, thereby improving both overall and disease free survival.

## Supplementary information

ESM 1 (DOC 363 kb)

## Data Availability

All data generated and analyzed are included in this article and its supplementary material files.

## References

[1] Benjanyan N, Weisdorf DJ, Logan BR, Wang H-L, Devine SM, de Lima M, Bunjes DW, Zhang M-J (2015). Survival of patients with acute leukemia relapsing after allogeneic hematopoietic cell transplantation: a Center for International Blood and Marrow Transplant Research study. Biol Blood Marrow Transplant.

[2] Kharfan-Dabaja MA, Labopin M, Polge E, Nishihori T, Bazarbachi A, Finke J, Stadler M, Ehninger G, Lioure B, Schaap N, Afanasyev B, Yeshurun M, Isaksson C, Maertens J, Calandon Y, Schmid C, Nagler A, Mohty M (2018). Association of second allogeneic hematopoietic cell transplant vs donor lymphpocyte infusion with overall survival in patients with acute myeloid leukemia relapse. JAMA Oncol.

[3] Lee SJ, Klein JP, Barrett AJ, Ringden O, Antin JH, Cahn JY, Carabasi MH, Gale RP, Giralt S, Hale GA, Ilhan O, McCarthy PL, Socié G, Verdonck LF, Weisdorf DJ, Horowitz MM (2002). Severity of chronic graft-versus-host diasease: association with treatment-related mortality and relapse. Blood.

[4] Dazzi F, Szydlo RM, Craddock C, Cross NC, Kaeda J, Chase A, Olavarria E, van Rhee F, Kanfer E, Apperley JF, Goldman JM (2000). Comparison of single-dose and escalating-dose regimens of donor lymphocyte infusion for relapse after allografting for chronic myeloid leukemia. Blood.

[5] Schmid C, Kuball J, Bug G (2021). Defining the role of donor lymphocyte infusion in high-risk hematologic malignancies. J Clin Oncol.

[6] de Lima M, Bonamino M, Vasconcelos Z, Colares M, Diamond H, Zalcberg I, Tavares R, Lerner D, Byington R, Bouzas L, da Matta J, Andrade C, Carvalho L, Pires V, Barone B, Maciel C, Tabak C (2001). Prophylactic donor lymphocyte infusions after moderately ablative chemotherapy and stem cell transplantation for hematological malignancies: high remission rate among poor prognosis patients at the expense of graft-versus-host disease. Bone Marrow Transplant.

[7] Ferrá C, Rodríguez-Luaces M, Gallardo D, Encuentra M, Martín-Henao GA, Peris J, Ancín I, Sarrá J, Berlanga JJ, García J, Granena A (2001). Individually adjusted prophylactic donor lymphocyte infusions after CD34-selected allogeneic peripheral blood stem cell transplantation. Bone Marrow Transplant.

[8] Dey BR, McAfee S, Colby C, Sackstein R, Saidman S, Tarbell N, Sachs DH, Sykes M, Spitzer TR (2003). Impact of prophylactic donor leukocyte infusions on mixed chimerism, graft-versus-host disease, and antitumor response in patients with advanced hematologic malignancies treated with nonmyeloablative conditioning and allogeneic bone marrow transplantation. Biol Blood Marrow Transplant.

[9] Krishnamurthy P, Potter VT, Barber LD, Kulasekararaj AG, Lim ZY, Pearce RM, de Lavallade H, Kenyon M, Ireland RM, Marsh JC, Devereux S, Pagliuca A, Mufti GJ (2013). Outcome of donor lymphocyte infusion after T cell-depleted allogeneic hematopoietic stem cell transplantation for acute myelogenous leukemia and myelodysplastic syndromes. Biol Blood Marrow Transplant.

[10] Eefting M, Halkes CJM, de Wreede LC, van Pelt CM, Kersting S, Marijt EWA, von dem Borne PA, Willemze R, Veelken H, Falkenburg JHF (2014). Myeloablative T cell-depleted alloSCT with early sequential prophylactic donor lymphocyte infusion is an efficient and safe post-remission treatment for adult ALL. Bone Marrow Transplant.

[11] Meyer RG, Britten CM, Wehler D, Bender K, Hess G, Konur A, Hartwig UF, Wehler TC, Ullmann AJ, Gentilini C, Uharek L, Huber C, Kolbe K, Herr W (2007). Prophylactic transfer of CD8-depleted donor lymphocytes after T-cell-depleted reduced-intensity transplantation. Blood.

[12] Yan C-H, Liu Q-F, Wu D-P, Zhang X, Xu L-P, Zhang X-H, Wang Y, Huang H, Bai H, Huang F, Ma X, Huang X-J (2017). Prophylactic donor lymphocyte infusion (DLI) followed by minimal residual disease and graft-versus-host-disease–guided multiple DLIs could improve outcomes after allogeneic hematopoietic stem cell transplantation in patients with refractory/relapsed acute leukemia. Biol Blood Marrow Transplant.

[13] Schmid C, Schleuning M, Schwerdtfeger R, Hertenstein B, Mischak-Weissinger E, Bunjes D, Harsdorf SV, Scheid C, Holtick U, Greinix H, Keil F, Schneider B, Sandherr M, Bug G, Tischer J, Ledderiose G, Hallek M, Hiddemann W, Kolb H-J (2006). Long-term survival in refractory acute myeloid leukemia after sequential treatment with chemotherapy and reduced-intensity conditioning for allogeneic stem cell transplantation. Blood.

[14] Gökbuget N, Stanze D, Beck J, Diedrich H, Horst H-A, Hüttmann A, Kobbe G, Kreuzer K-A, Leimer L, Reichle A, Schaich M, Schwartz S, Serve H, Starck M, Stelljes M, Stuhlmann R, Viardot A, Wendelin K, Freund M, Hoelzer D, on behalf of the German Multicenter Study Group for Adult Acute Lymphoblastic Leukemia (2012). Outcome of relapsed adult lymphoblastic leukemia depends on response to salvage chemotherapy, prognostic factors, and performance of stem cell transplantation. Blood.

[15] Legrand F, Le Floch AC, Granata A, Fürst S, Faucher C, Lemarie C, Harbi S, Bramanti S, Calmels B, El-Cheikh J, Chabannon C, Weiller PJ, Vey N, Castagna L, Blaise D, Devillier R (2017). Prophylactic donor lymphocyte infusion after allogeneic stem cell transplantation for high-risk AML. Bone Marrow Transplant.

[16] Jedlickova Z, Schmid C, Koenecke C, Hertenstein B, Baurmann H, Schwerdtfeger R, Tischer J, Kolb H-J, Schleuning M (2015). Long-term results of adjuvant donor lymphocyte transfusion in AML after allogeneic stem cell transplantation. Bone Marrow Transplant.

[17] Schmid C, Labopin M, Schaap N, Veelken H, Schleuning M, Stadler M, Finke J, Hurst E, Baron F, Ringden O, Bug G, Blaise D, Tischer J, Bloor A, Esteve J, Giebel S, Savani B, Gorin NC, Ciceri F, Mohty M, Nagler A (2019). Prophylactic donor lymphocyte infusion after allogeneic stem cell transplantation in acute leukemia – a matched pair analysis by the Acute Leukaemia Working Party of EBMT. Br J Haematol.

[18] Lutz C, Massenkeil G, Nagy M, Neuburger S, Tamm I, Rosen O, Dörken B, Arnold R (2008). A pilot study of prophylactic donor lymphocyte infusions to prevent relapse in adult acute lymphoblastic leukemias after allogeneic hematopoietic stem cell transplantation. Bone Marrow Transplant.

[19] Schmid C, Labopin M, Schaap N, Veelken H, Brecht A, Stadler M, Finke J, Baron F, Hurst E, Bug G, Ljungman P, Blaise D, Tischer J, Bloor A, Afanasyew B, Giebel S, Gorin N-C, Esteve J, Ciceri F, Savani B, Nagler A, Mohty M, Acute Leukaemia Working Party of the EBMT (2022). Long-term results and GvHD after prophylactic and preemptive donor lymphocyte Infusion after allogeneic stem cell transplantation (alloSCT) for acute leukemia. Bone Marrow Transplant.

[20] Buchholz S, Dammann E, Stadler M, Krauter J, Beutel G, Trummer A, Eder M, Ganser A (2012). Cytoreductive treatment with clofarabine/ara-C combined with reduced-intensity conditioning and allogeneic stem cell transplantation in patients with high-risk, relapsed, or refractory acute myeloid leukemia and advanced myelodysplastic syndrome. Eur J Haematol.

[21] Döhner H, Estey E, Grimwade D, Amadori S, Appelbaum FR, Büchner T, Dombret H, Ebert BL, Fenaux P, Larson RA, Levine RL, Lo Coco F, Naoe T, Niederwieser D, Ossenkoppele GJ, Sanz M, Sierra J, Tallman MS, Tien HF, Wei AH, Löwenberg B, Bloomfield C (2017). Diagnosis and management of acute myeloid leukemia in adults: 2017 European LeukemiaNet recommendations from an international expert panel. Blood.

[22] Bornhäuser M, Kienast J, Trenschel R, Burchert A, Hegenbart U, Stadler M, Baurmann H, Schäfer-Eckart K, Holler E, Kröger N, Schmid C, Einsele H, Kiehl MG, Hiddemann W, Schwerdtfeger R, Buchholz S, Dreger P, Neubauer A, Berdel WE, Ehninger G, Beelen DW, Schetelig J, Stelljes M (2012). Reduced-intensity conditioning versus standard conditioning before allogeneic haemopoietic cell transplantation in patients with acute myeloid leukaemia in first complete remission: a prospective, open-label randomised phase 3 trial. Lancet Oncol.

[23] Pfeifer H, Wassmann B, Bethge W, Dengler J, Bornhäuser M, Stadler M, Beelen D, Vucinic V, Burmeister T, Stelljes M, Schäfer-Eckhart K, Schwerdtfeger R, Lang E, Kubuschok B, Faul C, Dreger P, Kiani A, Brück P, Serve H, Schuld P, Hoelzer D, Gökbuget N, Ottmann OG, on behalf of the GMALL Study Group (2013). Randomized comparison of prophylactic and minimal residual disease-triggered imatinib after allogeneic stem cell transplantation for BCR–ABL1-positive acute lymphoblastic leukemia. Leukemia.

[24] Przepiorka D, Weisdorf D, Martin P, Klingemann HG, Beatty P, Hows J, Thomas ED (1995). 1994 Consensus conference on acute GvHD grading. Bone Marrow Transplant.

[25] Lee SJ, Vogelsang G, Gilman A, Weisdorf DJ, Pavletic S, Antin JH, Horowitz MM, Akpek G, Flowers ME, Couriel D, Martin PJ (2002). A survey of diagnosis, management, and grading of chronic GvHD. Biol Blood Marrow Transplant.

[26] Kaplan EL, Meier P (1958). Nonparametric estimation from incomplete observations. J Am Stat Assoc.

[27] Bertz H, Potthoff K, Finke J (2003). Allogeneic stem-cell transplantation from related and unrelated donors in older patients with myeloid leukemia. J Clin Oncol.

[28] Bortin MM, Rimm AA, Saltzstein EC (1973). Graft versus leukemia: quantification of adoptive immunotherapy in murine leukemia. Science.

[29] Weiden PL, Flournoy N, Thomas ED, Prentice R, Fefer A, Bruckner CD, Storb R (1979). Anti-leukemic effects of graft-versus-host disease in human recipients of allogeneic marrow grafts. N Engl J Med.

[30] Weiden PL, Sullivan KM, Flournoy N, Storb R, Thomas ED, Seattle Marrow Transplant Team (1981). Anti-leukemic effect of chronic graft-versus-host disease – contribution to improved survival after allogeneic marrow transplantation. N Engl J Med.

[31] Horowitz MM, Gale RP, Sondel PM, Goldman JM, Kersey J, Kolb HJ, Rimm AA, Ringdén O, Rozman C, Speck B (1990). Graft-versus-leukemia reactions after bone marrow transplantation. Blood.

[32] Song Y, Magenau J, Li Y, Braun T, Chang L, Bixby D, Hanauer DA, Chughtai KA, Gatza E, Couriel D, Goldstein S, Pawarode A, Reddy P, Riwes M, Connelly J, Harris A, Kitko C, Levine J, Yanik G, Parkin B, Choi SW (2015). FLT3 mutational status is an independent risk factor for adverse outcomes after allogeneic transplantation in AML. Bone Marrow Transplant.

[33] Yan CH, Liu DH, Liu KY, Xu LP, Liu YR, Chen H, Han W, Wang Y, Qin YZ, Huang XJ (2012). Risk stratification-directed donor lymphocyte infusion could reduce relapse of standard-risk acute leukemia patients after allogeneic hematopoietic stem cell transplantation. Blood.

[34] Sellmann L, Rabe K, Bünting I, Dammann E, Göhring G, Ganser A, Stadler M, Weissinger EM, Hambach L (2018). Diagnostic value of highly-sensitive chimerism analysis after allogeneic stem cell transplantation. Bone Marrow Transplant.

[35] Stadler M, Venturini L, Bünting I, Dammann E, Weissinger EM, Schwarzer A, Schultze-Florey C, Ehrlich S, Markel D, Lueck C, Gladysz A, Fröhlich T, Damrah N, Beutel G, Eder M, Ganser A, Hambach L (2022). Navigating preemptive and therapeutic donor lymphocyte infusions in advanced myeloid malignancies by high-sensitivity chimerism analysis. Front Oncol.

[36] Weissinger EM, Schiffer E, Hertenstein B, Ferrara JL, Holler E, Stadler M, Kolb HJ, Zander A, Zürbig P, Kellmann M, Ganser A (2007). Proteomic patterns predict acute graft-versus-host disease after allogeneic hematopoietic stem cell transplantation. Blood.

[37] Weissinger EM, Human C, Metzger J, Hambach L, Wolff D, Greinix HT, Dickinson AM, Mullen W, Jonigk D, Kuzmina Z, Kreipe HH, Schweier P, Böhm O, Türüchanow I, Ihlenburg-Schwarz D, Raad J, Durban A, Schiemann M, Koenecke C, Diedrich H, Holler E, Beutel G, Krauter J, Ganser A, Stadler M (2017). The proteome pattern cGvHD_MS14 allows early and accurate prediction of chronic GvHD after allogeneic stem cell transplantation. Leukemia.

[38] Levine JE, Braun TM, Harris AC, Holler E, Taylor A, Miller H, Magenau J, Weisdorf DJ, Ho VT, Bolanos-Meade J, Alousi AM, Ferrara JL, Blood and Marrow Transplant Clinical Trials Network (2015). A prognostic score for graft-versus-host disease based on biomarkers: a multicentre study. Lancet Haematol.

[39] Socié G, Zeiser R, Blazar B (2019). Immune biology of allogeneic hematopoietic stem cell transplantation.

[40] Hambach L, Vermeij M, Buser A, Aghai Z, va der Kwast T, Goulmy E (2008). Targeting a single mismatched minor histocompatibility antigen with tumor-restricted expression eradicates human solid tumors. Blood.

[41] Maury S, Lemoine FM, Hicheri Y, Rosenzwajg M, Badoual C, Cherai M, Beaumont J-L, Azar N, Dhedin N, Sirvent A, Buzyn A, Rubio M-T, Vigouroux S, Montagne O, Bories D, Roudot-Thoraval F, Vernant J-P, Cordonnier C, Klatzmann D, Cohen JL (2010). CD4^+^CD25^+^ regulatory T cell depletion improves the graft-versus-tumor effect of donor lymphocytes after allogeneic hematopoietic stem cell transplantation. Sci Transl Med.

[42] Maung KK, Chen BJ, Barak I, Li Z, Rizzieri DA, Gasparetto C, Sullivan KM, Long GD, Engemann AM, Waters-Pick B, Rowe Nichols K, Lopez R, Kang Y, Sarantopoulos S, Sung AD, Chao NJ, Horwitz ME (2021). Phase I dose escalation study of naive T-cell depleted donor lymphocyte infusion following allogeneic stem cell transplantation. Bone Marrow Transplant.

[43] Ciceri F, Bonini C, Stanghellini MT, Bondanza A, Traversari C, Salomoni M, Turchetto L, Colombi S, Bernardi M, Peccatori J, Pescarollo A, Servida P, Magnani Z, Perna SK, Valtolina V, Crippa F, Callegaro L, Spoldi E, Crocchiolo R, Fleischhauer K, Ponzoni M, Vago L, Rossini S, Santoro A, Todisco E, Apperley J, Olavarria E, Slavin S, Weissinger EM, Ganser A, Stadler M, Yannaki E, Fassas A, Anagnostopoulos A, Bregni M, Stampino CG, Bruzzi P, Bordignon C (2009). Infusion of suicide-gene-engineered donor lymphocytes after family haploidentical haemopoietic stem-cell transplantation for leukaemia (TK007 Trial): a non-randomised phase I-II study. Lancet Oncol.

[44] Weissinger EM, Borchers S, Silvani A, Provasi E, Radrizzani M, Beckmann IK, Benati C, Schmidtke J, Kuehnau W, Schweier P, Luther S, Fernandez-Munoz I, Beutel G, Ciceri F, Bonini C, Ganser A, Hertenstein B, Stadler M (2015). Long term follow up of patients after allogeneic stem cell transplantation and transfusion of HSV-TK transduced T-cells. Front Pharmacol.

[45] Tsirigotis P, Gkirkas K, Kitsiou V, Chondropoulos S, Athanassiades T, Thomopoulos T, Tsirogianni A, Stamouli M, Karagiannidi A, Siafakas N, Pappa V, Nagler A (2021). Repetitively administered low-dose donor lymphocyte infusion for prevention of relapse after allogeneic stem cell transplantation in patients with high-risk acute leukemia. Cancers.

