# Supplementary material for: The graft-versus-leukemia effect of prophylactic donor lymphocyte infusions after allogeneic stem cell transplantation is equally effective in relapse prevention but safer compared to spontaneous graft-versus-host disease
Source: Ann Hematol. 2023 Jul 25;102(9):2529–42. doi: 10.1007/s00277-023-05276-5 (PMC10444690; doi:10.1007/s00277-023-05276-5)
Supplement: Supplementary file 1 — (DOC 363 kb) [file 277_2023_5276_MOESM1_ESM.doc]

**Supplementary Material**

**Legends to Supplementary Table 2 and Figures**

Suppl.Table 2 Comparison of day 120- and day 160-landmark analyses

Suppl.Fig. 5 d +160 Landmark Analysis: comparison of proDLI patients to

patients without proDLI for *non-severe* GvHD

(A) Probability of Overall Survival (OS)

(B) Probability of Disease Free Survival (DFS)

(C) Probability of Relapse Incidence (RI)

(D) Probability of Non-Relapse Mortality (NRM)

Suppl.Fig. 6 d +160 Landmark Analysis of patients without subsequent clinically

relevant GvHD post day +160:

(A) Probability of Overall Survival (OS)

(B) Probability of Disease Free Survival (DFS)

(C) Probability of Relapse Incidence (RI)

(D) Probability of Non-Relapse Mortality (NRM)

Suppl.Fig. 7 d +160 Landmark Analysis: patients with a normal karyotype

(A) Probability of Overall Survival (OS)

(B) Probability of Disease Free Survival (DFS)

(C) Probability of Relapse Incidence (RI)

(D) Probability of Non-Relapse Mortality (NRM)

Suppl.Fig. 8 d +160 Landmark Analysis: FLT3-ITD positive patients

(A) Probability of Overall Survival (OS)

(B) Probability of Disease Free Survival (DFS)

(C) Probability of Relapse Incidence (RI)

(D) Probability of Non-Relapse Mortality (NRM)

**Supplementary Table 2 and Figures**

Suppl. Table 2: Comparison of day 120- and day 160-landmark analyses

day +120 day +160

landmark landmark

5y-OS (proDLI) 77% 77%

(spontaneous GvHD) 53% 54%

(contraindications) 45% 46%

*p (proDLI / no proDLI) <0.001 0.003*

5y-DFS (proDLI) 71% 67%

(spontaneous GvHD) 51% 53%

(contraindications) 42% 45%

*p (proDLI / no proDLI) 0.004 0.011*

5y-RI (proDLI) 25% 30%

(spontaneous GvHD) 30% 29%

(contraindications) 42% 39%

*p (proDLI+spontaneousGvHD*

*/ contraindications) 0.017 0.021*

5y-NRM (proDLI) 5% 5%

(spontaneous GvHD) 22% 18%

(contraindications) 24% 25%

*p (proDLI / no proDLI) 0.003 0.036*

5y-GvHD (proDLI) 21% 22%

post DLI (spontaneous GvHD) 20% 14%

(contraindications) 8% 7%

*p (proDLI / no proDLI) not signif. not signif.*

Supplementary Figure 5

d +160 Landmark Analysis: comparison of proDLI patients to **patients without proDLI for *non-severe* GvHD**

(A) Probability of Overall Survival (**OS**)


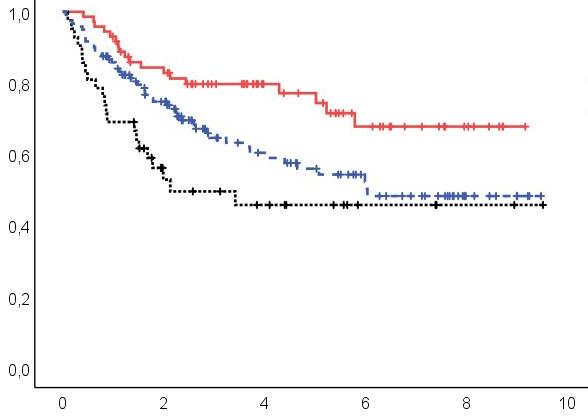
years

*p = 0.005*

OS

proDLI

no proDLI (*non-severe* GvHD)

no proDLI (contraindications)

d +160

(B) Probability of Disease Free Survival (**DFS**)


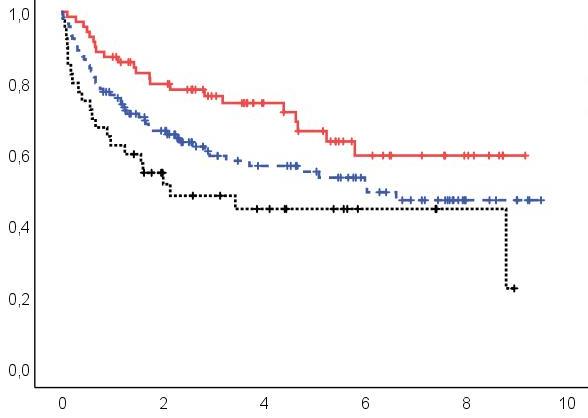
 years

DFS

d +160

*p = 0.016*

proDLI

no proDLI (*non-severe* GvHD)

no proDLI (contraindications)

Supplementary Figure 5

d +160 Landmark Analysis:

(C) Probability of Relapse Incidence (**RI**)


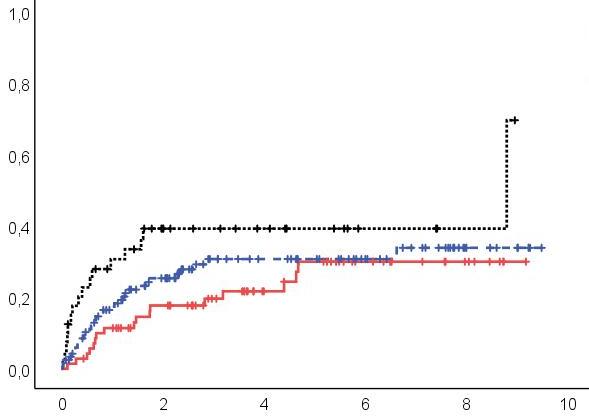
years

RI

d +160

no proDLI (contraindications)

no proDLI (*non-severe* GvHD)

proDLI

*n.s.*

*p = 0.033*

(D) Probability of Non Relapse Mortality (**NRM**)


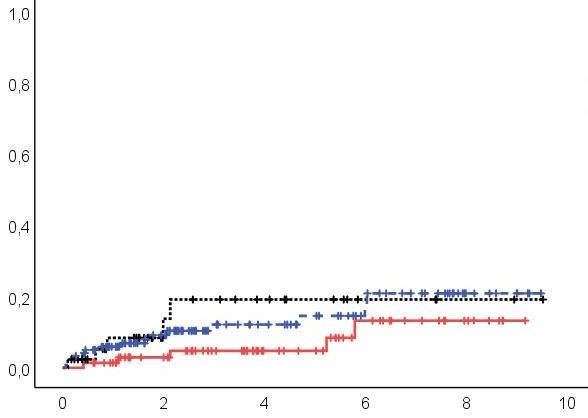
 years

NRM

d +160

*n.s.*

no proDLI (*non-severe* GvHD)

no proDLI (contraindications)

proDLI

Supplementary Figure 6

d +160 Landmark Analysis of **patients without subsequent clinically relevant GvHD post day +160**

(A) Probability of Overall Survival (**OS**)


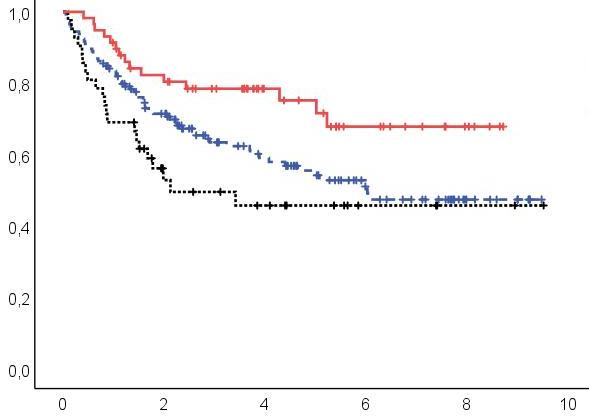
years

*p = 0.011*

OS

proDLI

no proDLI (GvHD)

no proDLI (contraindications)

d +160

(B) Probability of Disease Free Survival (**DFS**)


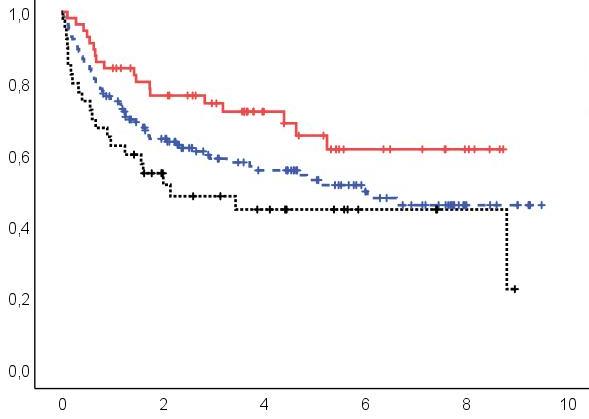
 years

DFS

d +160

*p = 0.031*

proDLI

no proDLI (GvHD)

no proDLI

(contraindications)

Supplementary Figure 6

d +160 Landmark Analysis:

(C) Probability of Relapse Incidence (**RI**)


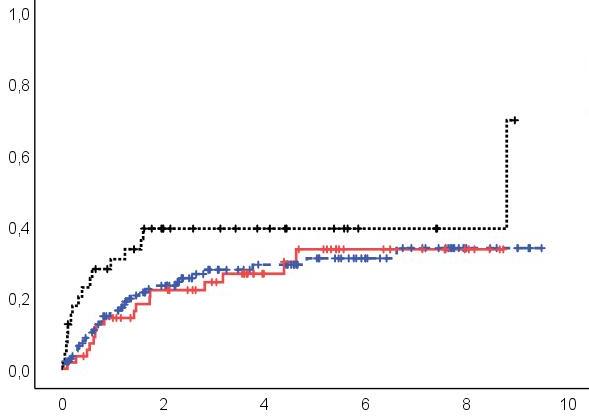
years

RI

d +160

no proDLI (contraindications)

no proDLI (GvHD)

proDLI

*n.s.*

*p = 0.040*

(D) Probability of Non Relapse Mortality (**NRM**)


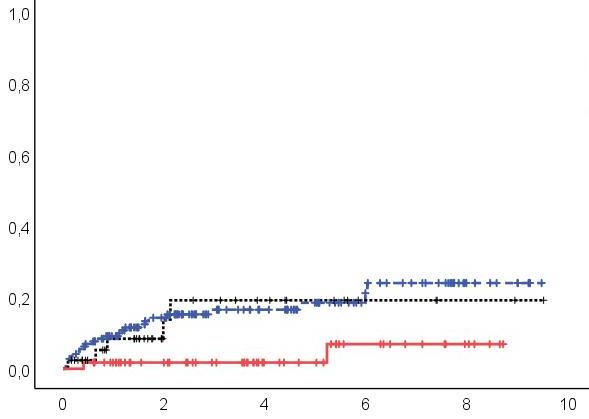
 years

NRM

d +160

*p = 0.012*

no proDLI (GvHD)

no proDLI (contraindications)

proDLI

Supplementary Figure 7

d +160 Landmark Analysis: **patients with a normal karyotype**

(A) Probability of Overall Survival (OS)


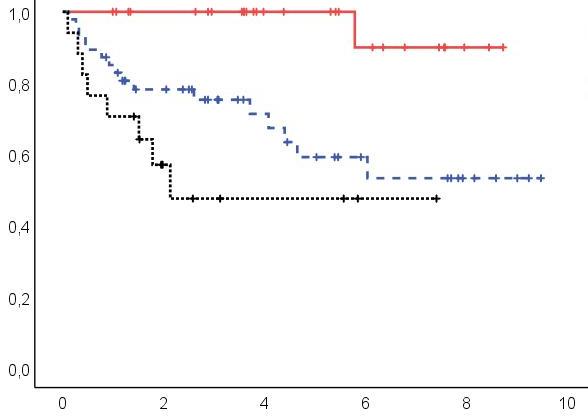
years

OS

proDLI

no proDLI (GvHD)

no proDLI (contraindications)

d +160

(B) Probability of Disease Free Survival (DFS)


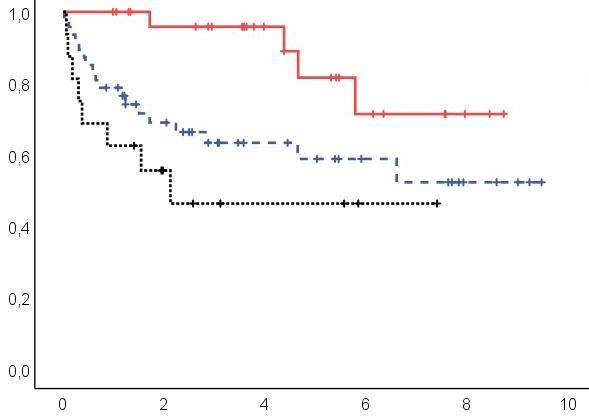
 years

DFS

d +160

proDLI

no proDLI (GvHD)

no proDLI (contraindications)

Supplementary Figure 7

d +160 Landmark Analysis:

(C) Probability of Relapse Incidence (RI)


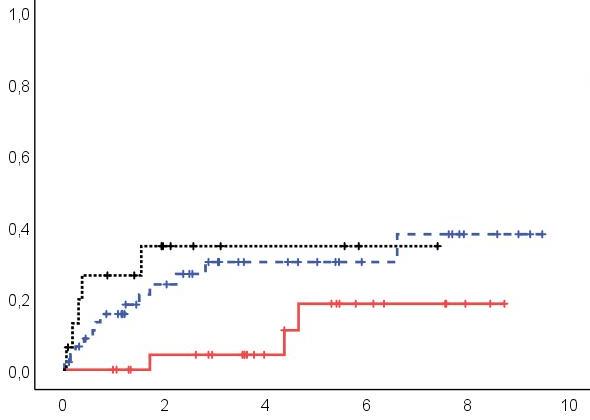
years

RI

d +160

no proDLI (contraindications)

no proDLI (GvHD)

proDLI

(D) Probability of Non-Relapse Mortality (NRM)


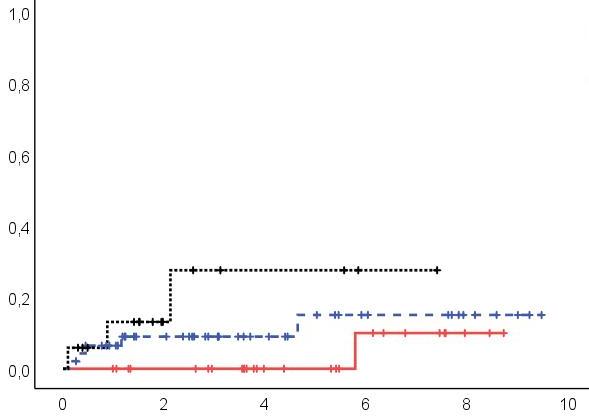
 years

NRM

d +160

no proDLI (contraindications)

no proDLI (GvHD)

proDLI

Supplementary Figure 8

d +160 Landmark Analysis: **FLT3-ITD positive patients**

(A) Probability of Overall Survival (OS)


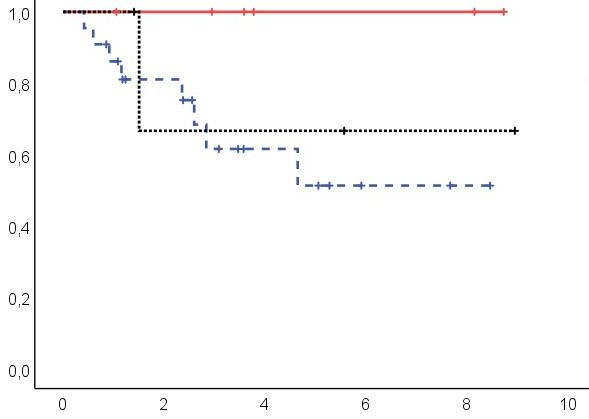
years

OS

proDLI

no proDLI (contraindications)

no proDLI (GvHD)

d +160

(B) Probability of Disease Free Survival (DFS)


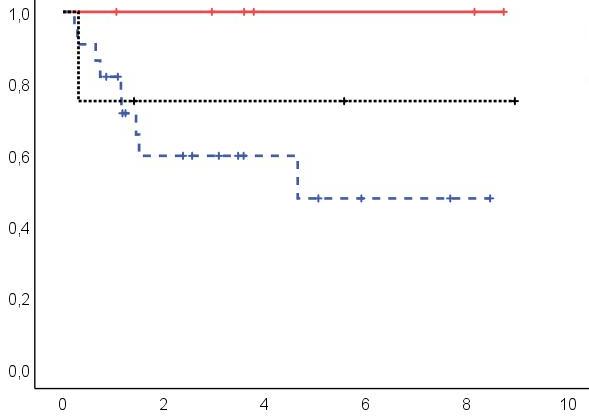
 years

DFS

d +160

proDLI

no proDLI (contraindications)

no proDLI (GvHD)

Supplementary Figure 8

d +160 Landmark Analysis:

(C) Probability of Relapse Incidence (RI)


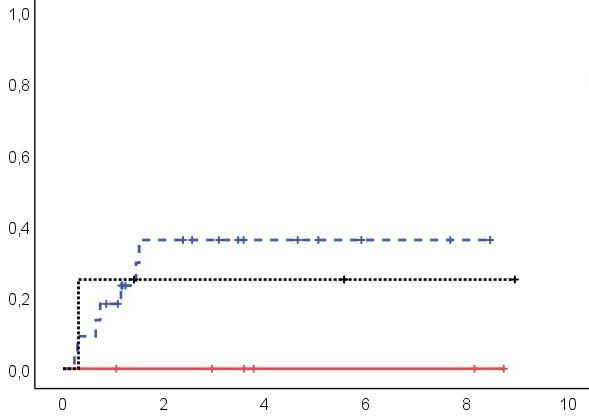
years

RI

d +160

no proDLI (GvHD)

no proDLI (contraindications)

proDLI

(D) Probability of Non-Relapse Mortality (NRM)


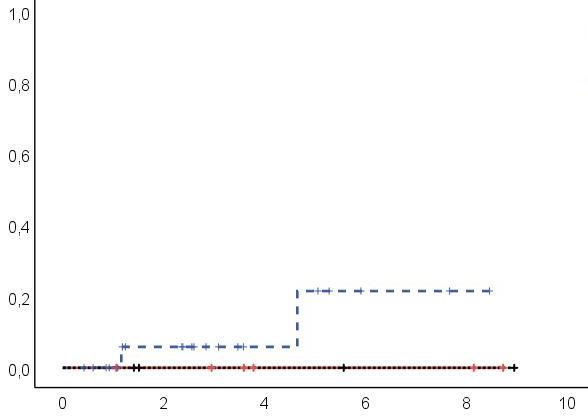
 years

NRM

d +160

no proDLI (GvHD)

no proDLI (contraindications) proDLI
